# Enhancing Extended Reality Technology for Neuromusculoskeletal Rehabilitation: Recommendations for the Development of Clinically Relevant Serious Games

**DOI:** 10.3390/jpm16020111

**Published:** 2026-02-12

**Authors:** Adrien Moevus, An Kateri Vu, Karla Rodrigues Soares Menezes, Mindy F. Levin, Dahlia Kairy

**Affiliations:** 1 École de Réadaptation, Faculté de Médecine, Université de Montréal, Montreal, QC H3C 3J7, Canada; adrien.moevus@umontreal.ca (A.M.); karla.soares@umontreal.ca (K.R.S.M.); 2 Center for Interdisciplinary Research in Rehabilitation of Greater Montreal (CRIR), Montreal, QC H3S 1M9, Canada; an.kateri.vu@usherbrooke.ca (A.K.V.); mindy.levin@mcgill.ca (M.F.L.); 3 Institut Universitaire sur la Réadaptation en Déficience Physique de Montréal (IURDPM), Montreal, QC H3S 2J4, Canada; 4 Faculté de Médecine et des Sciences de la Santé, Université de Sherbrooke, Sherbrooke, QC J1K 2R1, Canada; 5 School of Physical and Occupation Therapy, McGill University, Montreal, QC H3A 0G4, Canada

**Keywords:** virtual reality, augmented reality, extended reality, personalization, neuromusculoskeletal rehabilitation, serious games, motor learning, gamification, exergames, motor control, physical therapy

## Abstract

**Background**: Although traditional rehabilitation methods are effective in promoting recovery for patients with disabilities, some approaches can involve repetitive tasks, making it challenging to maintain high patient engagement and adherence. This can impact the amount of therapy patients receive, which can sometimes limit their overall recovery potential, particularly given constraints in healthcare resources. Extended reality (XR) technologies, which include virtual reality (VR) and augmented reality (AR), offer promising benefits to personalize care and enhance rehabilitation and engagement by increasing motivation and engagement through interactive and immersive environments. Despite these promising advantages, their successful integration in clinical practice has remained limited, partly due to lack of early involvement of clinicians and end-users in the development process. **Objective**: We aim to provide recommendations for XR rehabilitation technology development, including researchers and industry professionals, to foster more personalized, adoptable and effective tools for patients with neuromusculoskeletal disorders in a clinical setting. **Methods**: Principles from motor control and game theory are used to describe key features and recommendations for XR rehabilitation technology development to optimize rehabilitation applications in a clinical setting. These recommendations stem from established motor learning and game design principles, a state-of-the-art narrative review of emerging XR rehabilitation literature (2015–2025) and insights from the Ensemble! Program, a living lab where clinicians, researchers, and patients collaborate to explore emerging technologies, including but not limited to serious games using XR technologies. **Results**: Key design recommendations include strategies for supporting patient motivation, adjusting game difficulty, providing feedback and handling data collection. **Conclusions**: Integrating motor control and game theory principles into XR rehabilitation technology can help optimize its therapeutic effectiveness and clinical applicability for patients with neuromusculoskeletal conditions. By addressing clinician and patient needs early in the development process, these technologies can be better tailored to meet therapeutic goals and facilitate broader adoption in clinical practice.

## 1. Introduction

Serious games for rehabilitation are exergames designed with the primary goal of facilitating physical recovery rather than patient entertainment [1]. The use of exergames for rehabilitation is gaining more recognition with the demonstration of improved daily living functions in patients with neurological disorders [2]. In recent years, the field of serious games has expanded to include various immersive and non-immersive technologies such as active video games (AVGs), virtual reality (VR) games, and augmented reality (AR) games. VR exergames are created in an immersive or semi-immersive, computer-generated environment in which users can interact with various objects or tasks via specialized equipment such as controllers. In contrast, augmented reality (AR) exergames add computer-generated elements onto the real-world environment to enhance the user’s experience through multiple sensory modalities: visual elements (such as virtual 3D objects, images, or avatars), auditory elements (such as sounds, music, or voice instructions), and tactile elements (such as haptic vibrations or force feedback) [3]. Both VR and AR fall under the broader category of extended reality (XR) [3]. Each of these modalities which allow more personalized interventions offers unique advantages for physical rehabilitation [4,5,6].

Immersive environments may visually and auditorily engage users via head-mounted displays (HMDs) and spatial audio to enhance the sense of “presence”, allowing users to feel as if they are physically present within the virtual space and to interact as they would in real life. In contrast, non-immersive environments are usually projected onto standard screens (e.g., computer monitors or televisions). Both types of systems may use similar technologies such as motion tracking, controllers, or camera-based gesture tracking [7]. AR serious games can also enhance presence by anchoring virtual elements to the real physical environment, allowing patients to see and interact with digital objects within their own familiar surroundings. This connection to the real world may reduce disorientation and increase engagement, as patients can maintain awareness of their physical space while performing therapeutic tasks. This diverse landscape of extended reality (XR) serious games presents promising opportunities for personalized rehabilitation approaches that cater to the unique needs of patients. This commentary paper is based on relevant emerging literature and our team’s recent experiences with XR technologies as examples to provide recommendations that can help enhance the development of extended reality (XR) rehabilitation technology.

Enriched training environments may enhance neuroplasticity, a critical factor in recovery [5] after neurological injury or disease. Both VR and AR offer activities that can be tailored to individual rehabilitation needs; however, AR allows patients to interact with their real-world environment, providing a safer and more relevant context for tasks like ambulation, as patients remain aware of their physical surroundings while training [8,9]. Devices that switch between VR and AR modes are particularly promising for clinical use, as they offer the flexibility of choosing between immersive simulations when needed and real-world interaction with simulated elements that can facilitate a smoother transition to daily activities for patients [10].

Despite the potential benefits, the adoption of these technologies in clinical settings has been slow [11]. A recent scoping review by Alt Murphy et al. [11] identified several barriers to adoption, including patients’ perceived effort required to learn and implement new technologies, the need for user-friendly design, and difficulties in integrating these technologies with existing healthcare systems. Indeed, uptake among clinicians has been hampered by factors such as time constraints, the need for a supportive organizational culture, and the lack of clinician and patient involvement in both the design and evaluation of these technologies. Without early involvement and feedback from end-users, new tools risk being poorly adapted to clinical workflows and rehabilitation priorities. In short, for successful adoption, XR technologies must support rehabilitation goals without adding to the clinical workload [11]. To overcome these barriers, Alt Murphy et al. [11] recommended that XR technology developers should engage clinicians and patients early in the development process to ensure that new technologies address real clinical needs and are intuitive for intended users. However, securing active participation from clinicians, who are often already overburdened, remains a challenge.

### 1.1. Approach and Methods

This commentary integrates motor learning and game design principles with insights from a state-of-the-art narrative review of emerging XR rehabilitation literature and observations from a living lab implementation study.

#### 1.1.1. Motor Learning and Game Design Principles

Recommendations are grounded in theoretical principles of motor control, neuroplasticity, and game design. Specifically, we draw upon Kleim and Jones’ 10 principles of experience-dependent neural plasticity [12], which emphasize repetition, intensity, specificity, and salience in driving neuroplastic change; Guadagnoli and Lee’s Challenge Point Framework [13], which guides optimal difficulty calibration to balance learning and motivation; Wulf and colleagues’ OPTIMAL theory [14], which highlights the role of autonomy, external focus, and enhanced expectancies in motor learning; and Chiviacowsky and Wulf’s findings on feedback timing and content [15], demonstrating that feedback after successful trials enhances learning. From game design theory, we incorporate principles of intrinsic motivation [16], loss aversion and difficulty balancing, and gamification elements that sustain engagement [17]. These foundational frameworks provide evidence-based guidance on motivation, difficulty calibration, feedback delivery, and task specificity in rehabilitation contexts.

#### 1.1.2. Narrative Review of Emerging XR Applications

To identify innovative applications and contemporary challenges in XR rehabilitation technology, we conducted a state-of-the-art narrative review of recent XR literature. State-of-the-art narrative reviews are particularly appropriate for rapidly evolving fields, as they summarize current understanding, describe how knowledge has evolved, and identify future research directions. The XR-focused search included PubMed and Google Scholar queries for recent publications (2015–2025) addressing virtual reality, augmented reality, or non-immersive exergames for neuromusculoskeletal rehabilitation, with emphasis on clinical implementation barriers and emerging technical capabilities [6,7,8,9,10,11,12,18,19,20,21,22,23,24,25]. As is consistent with narrative review methodology, this search was targeted rather than exhaustive, aiming to capture representative and pivotal literature addressing current innovations and challenges in XR rehabilitation.

#### 1.1.3. Ensemble! Program’s Technological Park

Since 2023, our team has worked in a unique collaborative environment, the Ensemble! Program’s technological park, a living lab set in the Gingras-Lindsay Rehabilitation Institute of Montreal (IRGLM, QC, Canada). A living lab is a multidisciplinary environment of collaboration, knowledge exchange, co-creation, and real-world testing among researchers, clinicians and patients [26,27].

The Ensemble! Program’s living lab technology park serves as an environment where various emerging technologies are made accessible to patients while allowing researchers to gather valuable feedback from clinicians and patients. These technological activities include an upper limb training apparatus called the “exerciseur”, which is a seated device in which patients push and pull against robot-assisted metal arms to meet a visual target while a computer measures upper-limb strength, a “recumbent bicycle”, which allows patients to engage in assisted and resisted cycling exercises to improve endurance and lower limb function, and a “split belt treadmill” equipped with a safety harness and dual belts, which allows for gait analysis and training, weight distribution assessment, and the simulation of balance perturbations during gait to safely practice falls. In addition, the living lab includes XR serious games, which are body-tracking games designed to train coordination and gross motor control in an engaging and motivating manner.

Practical insights from the Ensemble! Program living lab complemented the literature synthesis. The living lab operates as part of an ongoing implementation study where clinicians, researchers, and patients collaborate to explore emerging technologies in real-world clinical settings. Between May 2024 and August 2024, 21 inpatients (18 M/3 W, ages 27–80 years, mean 54.90 ± 3.73) with neuromusculoskeletal impairments (spinal cord injuries (*n* = 11), strokes (*n* = 8) and amputees (*n* = 2)), used XR serious games as a supplement to standard rehabilitation. Patients were referred by treating therapists and participated in two 40-min sessions per week. For the purpose of this article, observations and data were collected informally during regularly scheduled patient training sessions to inform reflections on technology design. Qualitative observations from clinician feedback were reviewed thematically to identify recurring points related to usability, engagement, and clinical integration challenges.

The recommendations presented in this commentary (Table 1) synthesize foundational motor learning principles with emerging XR innovations identified through the narrative review and contextualized by real-world implementation observations from the living lab. This integrative approach ensures that theoretical principles are grounded in practical clinical constraints and contemporary technological capabilities. The recommendations are intended to serve as a tool for developers and users (academic, industry, clinical) to ensure XR exergames align with core physical rehabilitation principles and clinical needs, as well as to serve as a starting point for further reflections regarding ongoing XR exergame development and use.

## 2. Principles to Optimize Physical Rehabilitation Using XR Serious Games

Developing an effective serious game for physical rehabilitation demands an adherence to key motor learning principles. These principles include motivation, difficulty level, feedback type and delivery schedule, and task specificity [12,16].

### 2.1. Motivation and Purpose

Motivation is a critical factor in the success of any rehabilitation program. For patients to engage fully in rehabilitation, they must not only understand the purpose of the exercises but also be engaged in the tasks at hand. Research suggests that well-designed serious games can significantly enhance patient motivation compared to standard rehabilitation methods [2,28]. This improvement is attributed to the incorporation of game design principles such as immediate feedback, reward systems, achievable goals, and fostering a sense of accomplishment [15,29]. Cognitive and emotional engagement are essential, as they directly influence neuroplasticity and as such the effectiveness of rehabilitation exercises [12].

Incorporating elements of gamification, such as scoring systems, contextual narratives, and real-time feedback, can transform otherwise routine exercises into meaningful and engaging experiences. For example, rather than having a patient perform a repetitive shoulder abduction exercise, a game can allow the player to control a spaceship, raising and lowering their arm to collect fuel and earn points. Adding a simple narrative with good feedback and a reward system can improve a patient’s engagement [15,30].

Gamification activates the brain’s reward system, primarily mediated by dopamine [29]. In rehabilitation, when a patient experiences a sense of achievement or pleasure from completing a task, dopamine is released, enhancing neural pathways involved in motor control [12,29]. This reward-based feedback loop transforms repetitive exercises into rewarding activities, encouraging continued participation and improving motor recovery outcomes [28,30].

### 2.2. Loss Aversion

Loss aversion, the psychological principle that people are more motivated to avoid losses than to acquire equivalent gains, is a powerful tool in game design [16]. Traditional games typically reward players for correct actions, but incorporating loss aversion, such as penalty systems for mistakes, can further enhance motivation. This mechanism motivates players to maintain focus and accuracy to avoid losing their progress. However, these mechanics must be carefully calibrated to avoid frustration, particularly for patients with cognitive impairments. Penalties should be mild and recoverable (e.g., temporarily losing a visual streak indicator rather than permanent point deductions), and thresholds should be adjusted to match patient capabilities [1]. Overly punitive systems may discourage rather than motivate engagement [13,16].

In physical rehabilitation games, similar mechanics can be employed to reinforce correct movements and discourage compensatory behaviors, i.e., inefficient or even harmful movement patterns (e.g., joint overuse or abnormal muscle loading). AR rehabilitation exergames have demonstrated effective use of visual feedback mechanisms to guide patient performance. For instance, in AR games designed for children with neurological impairments, tiles illuminate in blue to indicate targets, then change to green with accompanying sounds when patients correctly reach them, providing immediate multimodal confirmation of proper movement execution [25]. When errors occur, the absence of these positive visual and auditory cues signals the need for correction without explicit instruction, supporting implicit motor learning [21]. These progressive visual reward systems align with loss aversion principles by creating a sense of maintained achievement through consecutive successes [12].

## 3. Modulating Difficulty Level in Physical Rehabilitation XR Serious Games

### 3.1. Challenge and Dynamic Difficulty

In serious games for physical rehabilitation, appropriately calibrated challenges sustain motivation and engagement and support motor learning [13]. The task’s difficulty should match the user’s current skill, neither too easy nor too hard, with adaptive progression to maintain a productive zone for practice [17]. The optimal level is set empirically by adjusting task difficulty and feedback based on observed performance, reflecting the dynamic interaction of difficulty, skill, and feedback over time [17].

Game theory emphasizes the importance of perceiving challenges as fair so that success is achievable through skill improvement. Unfair challenges, where success feels unattainable, can lead to frustration and disengagement. Therefore, the ability to calibrate and adjust the difficulty level is crucial in game design, especially in therapeutic settings, where maintaining patient motivation for rehabilitation exercises is important [16].

Dynamic difficulty adjustment is a method to maintain an optimal challenge level throughout the game [17]. Determining an optimal challenge level is generally done empirically by trial and error while considering the user’s feedback and performance. By automatically adapting the game difficulty in response to the player’s performance, the game remains neither too easy nor too difficult, thereby enhancing the player’s enjoyment, motivation, and engagement [13,17]. Hence, dynamic difficulty adjustment can be particularly beneficial for rehabilitation applications as it allows the game to remain challenging while being tailored to the patient’s current capabilities.

While automated difficulty adjustment can maintain optimal challenge during gameplay, it should operate within clinician-defined boundaries [11]. Clinicians need to establish acceptable ranges for automated modifications (e.g., target size from 5–15 cm, movement speed from slow to moderate) to ensure that real-time adjustments remain therapeutically appropriate and do not inadvertently encourage compensatory movements or exceed safe movement limits [5,32]. This hybrid approach, combining automated real-time adaptation with clinician oversight, balances responsiveness to patient performance with clinical safety and therapeutic goals.

To facilitate clinician control, a companion app, a software that runs on a different device than the main application, can provide an accessible interface for adjusting difficulty parameters. However, developers should be mindful of clinical time constraints; parameter adjustment interfaces should be intuitive enough that clinicians with varying levels of technical expertise can make modifications quickly [11,18]. Features such as preset templates, slider-based controls with visual previews, and single-tap difficulty scaling can help ensure that game customization enhances rather than burdens clinical workflow.

### 3.2. Defining Difficulty in Physical Rehabilitation Games

The concept of difficulty in physical rehabilitation games can differ significantly from traditional video games. For example, while increasing the speed of a task might typically raise the difficulty level in a conventional game, such an approach may not be suitable for rehabilitation exercises focused on endurance or precision. Instead, the difficulty in a rehabilitation context may need to be tailored to enhance specific physical capabilities without compromising the overall therapeutic goals.

Fitts [33] described a reaching performance task used to model and predict skilled upper limb movement. In this task, the difficulty of movement increases when the target is placed farther from the initial hand position, target size is reduced and movement speed is increased. Also, for reaching tasks, the placement of targets at varying distances and positions (e.g., contralateral, midline, ipsilateral) within the workspace can encourage the coordinated use of different combinations of arm and trunk segments, thereby increasing the complexity of the task [32]. For developers, incorporating adjustable target positions and sizes is a way to increase a game’s therapeutic value. Therefore, when adding predefined difficulty levels, developers should consider that distance from the body, target size, speed of movement, and the number of joints involved all contribute to the movement’s difficulty.

## 4. Considerations for Feedback in Physical Rehabilitation XR Serious Games

### 4.1. Delivery of Extrinsic Feedback

Virtual reality enables flexible delivery of extrinsic feedback, information provided by an external source about movement performance, which can enhance motor learning in patients with neurological impairments [14,15]. Extrinsic feedback is provided by an external source, such as a therapist’s verbal cues, visual biofeedback, or tactile signals from a device [14]. Patients recovering from stroke or traumatic brain injury often have diminished capacity to detect and correct movement errors independently, making extrinsic feedback crucial for reinforcing proper motor patterns and accelerating rehabilitation progress [14,32].

Various methods can be used to deliver extrinsic feedback, and choosing the appropriate type for the situation is critical. Feedback not only guides the patient’s movements but also influences their motivation. Extrinsic feedback is usually categorized into two types: knowledge of performance (KP) and knowledge of results (KR). KP focuses on the parameters of a movement, how the movement was performed, while KR reports the success or failure of the movement’s outcome. For example, if a patient successfully raises their arm to a certain height but does so with excessive trunk flexion, KR could acknowledge the achievement of reaching the height, whereas KP could provide information about the compensatory movement, prompting the patient to correct their technique [14,15].

Research suggests that for novices, a combination of prescriptive KP (instructions on how to improve movement) and KR yields the best outcomes [14]. This combined approach helps patients understand both the success of their actions and the specific adjustments needed for improvement [14]. However, there are also potential downsides of relying heavily on instructions. Overly prescriptive feedback can lead to a dependence on external cues, which might inhibit the development of internal error detection and self-regulation. This can hamper the natural exploratory process that underlies motor learning, particularly as learners become more skilled [14,15]. In some instances, too much detailed instruction may overload the learner’s cognitive resources, reducing their ability to integrate and adapt feedback effectively [34].

In the living lab context of the technology park, a combination of KP and KR can be provided as feedback. However, feedback mechanisms in some VR-based rehabilitation games often prioritize KR over KP. Despite promising research outcomes for VR in rehabilitation, Levin et al. [5] highlighted that the ideal combinations and types of feedback most effective to enhance upper limb motor function remain unclear. This underscores the need for further investigation into feedback strategies to maximize therapeutic benefits [5].

### 4.2. Negative Versus Positive Feedback

Feedback can be either positive, affirming correct actions, or negative, highlighting errors. In the context of KR, providing feedback after successful task completion is more effective than offering feedback following poor performance [13]. This approach encourages patients by reinforcing their successes, which can boost motivation and confidence [25]. Negative feedback about excessive trunk movement, for example (i.e., KP), might be used to provide information about unwanted compensatory movements, while positive feedback may be used to reinforce acceptable postures and techniques.

The mechanisms of loss aversion, such as the combo mechanism or streak tracker discussed earlier, where a score multiplier resets every time the patient makes an error but builds up as they continuously succeed, are good examples of both positive and negative feedback. The combination of positive feedback (rewarding progress) and negative feedback (correcting errors) within the framework of loss aversion has the potential to create a powerful, balanced system that enhances engagement, motivation, and motor learning in rehabilitation settings [13]. However, feedback strategies should be adapted to the patient population. For patients with neurological impairments, such as stroke, feedback should be simplified to avoid cognitive overload, for example, using a single auditory cue for compensatory movements with visual summaries provided only after task completion [5,14]. Conversely, patients with musculoskeletal conditions without cognitive deficits may benefit from richer, concurrent visual feedback that allows real-time movement adjustment [34].

### 4.3. Providing Feedback in Extended Reality

Providing feedback in XR serious games in rehabilitation poses distinct challenges to ensure optimal use in physical rehabilitation. One potential issue is sensory overload, as virtual and augmented environments can easily overwhelm patients with excessive visual or auditory information, making it difficult for them to focus on critical feedback. To mitigate this, feedback should be minimal and contextually integrated into the virtual or augmented environment, such as through subtle visual cues or brief auditory signals that blend into the task [5]. Particularly for negative feedback about the use of compensatory movements, this should be done in a way that is not disruptive to the task [5]. For example, when a patient performs excessive trunk flexion, a therapist can explain beforehand that a small unpleasant sound will signal this error, allowing the patient to self-correct without explicit instruction during the task. This approach supports implicit learning by prompting the patient to evaluate and adjust their own posture rather than relying on detailed external cues [5]. It is especially important to consider how not to disrupt the task for patients with cognitive deficits, as excessive instructional feedback can overload cognitive resources and reduce the ability to integrate and adapt information effectively [14]. For these patients, feedback strategies should prioritize simplicity and minimize simultaneous demands on attention, for example by using a single subtle auditory cue rather than multiple sources of information, thereby supporting motor learning without overwhelming cognitive capacity [5,14].

Simple graphics or performance data can also be displayed within the XR interface, for example on a virtual scoreboard at the end of each game session or as a small on-screen indicator during gameplay, to show how well the person has performed, such as the percentage of successful task completion or the number of specific movements completed [15]. These visual summaries provide knowledge of results that can enhance motivation by making progress tangible and offering clear goals for improvement [15].

Physical rehabilitation games often feature various activities that do not necessarily have a link between them. An effective way to provide overall feedback and a sense of continuous progression to users is to create an overall scoring system, perhaps via experience points and levels that increase every time a task is completed. This can be made more specific, for example, by having levels for various body parts or types of tasks, such that every game involving the arms would increase the experience points for the arms level or balance level, and so on. This not only may add to the sense of purpose and sustained engagement, but may also help users track their overall progress across diverse rehabilitation activities [13].

### 4.4. Rehabilitation Setting

When developing physical rehabilitation exergames, determining the appropriate setting in which the game will be played is important in order to provide appropriate feedback. The exergame may be designed for various settings, for example clinic-based sessions or home use with or without supervision.

If the game is designed for supervised use, perhaps in a clinic or through remote telerehabilitation, developers have several options. The game can be integrated into a clinical setting, used with remote supervision via a clinician monitoring the patient’s movements on a computer, or employed as a hybrid tool where initial sessions are supervised, followed by unsupervised play once the clinician is confident in the patient’s ability to perform the movements correctly. Regular check-ins can then be scheduled to monitor progress and make necessary adjustments to ensure the game remains motivating and is at the appropriate difficulty level for the patient.

For games intended to be played without supervision, perhaps at home, mechanisms that ensure that the patient performs movements correctly and receives real-time, accurate feedback should be in place. Developers should consider practical home deployment challenges, such as variable lighting conditions, limited space, and the need for user-friendly calibration procedures that patients can complete independently [11,31]. As highlighted in Kleim and Jones [12], patients recovering from brain injuries may develop compensatory strategies that, while easier to perform, are less effective for long-term rehabilitation. Exergames should therefore incorporate precise motion tracking and robust analytics to monitor specific indicators of movement quality (such as joint angles, endpoint movement smoothness, and the extent of compensatory trunk displacement). Clinicians can use this data to assess whether patients are exhibiting compensatory behaviors that could lead to inefficient or even harmful movement patterns (e.g., joint overuse or abnormal muscle loading) and intervene by modifying task difficulty or providing corrective feedback (such as the faded KP and KR feedback strategies described earlier) to guide patients toward more effective movement patterns.

Beyond ensuring correct movement execution, patient safety deserves particular emphasis for unsupervised home-based use. Patients with balance impairments, reduced proprioception, or cognitive deficits face heightened fall and injury risks when using immersive XR systems without supervision [11,18]. Essential safety features include virtual boundaries with real-time collision alerts, automated fatigue monitoring to prevent overexertion, easily accessible emergency pause functions (e.g., voice commands or large buttons), and clear exit protocols [8,18]. For patients with cognitive impairments, simplified visual or auditory safety cues can reduce confusion and support safer independent use [14,34]. Integrating these mechanisms early in development ensures that home-based XR rehabilitation is both effective and safe for vulnerable populations [12,31].

The choice of setting should be guided by the specific needs of the patient population and the goals of the rehabilitation program. A setting like the Ensemble! Program living lab offers a valuable opportunity for developers to gather ongoing feedback from both clinicians and users. This feedback can be instrumental in refining the exergame’s design and ensuring that it meets the intended needs while maximizing its therapeutic effectiveness.

### 4.5. Important Features for Clinical Adoption

While this section does not aim to provide an exhaustive list of features, it focuses on four aspects that have emerged as important for the adoption of serious games by clinicians in physical rehabilitation: data analytics, game adjustability, a companion application and data confidentiality. These priorities were identified through feedback from clinicians and patients in the Ensemble! Program living lab, as well as alignment with barriers to technology adoption documented in the literature [11,26]. These features are fundamental to ensuring that the exergames are not only engaging but also clinically useful and adaptable to the needs of diverse patient populations.

### 4.6. Data Analytics and Visualization

Incorporating robust data analytics into serious games is paramount for both clinical effectiveness and research validation. A well-designed rehabilitation game should go beyond merely engaging patients. It should provide detailed insights into the patient’s movements, such as the frequency, quality (e.g., movement smoothness and patterns), and consistency (e.g., steadiness in speed or range of motion) of the exercises performed. This type of data is needed for clinicians to assess the patient’s progress, identify compensatory behaviors, and pinpoint areas requiring additional focus.

Specifically, XR rehabilitation games should track and report key kinematic metrics that are clinically meaningful and well-validated. Movement smoothness is a fundamental indicator of motor recovery and should be quantitatively assessed [35], as it has demonstrated sensitivity in detecting motor improvements in neurological populations. Joint angles and range of motion (ROM) should be monitored to verify that patients are achieving therapeutic movement targets [36]. Compensatory movement patterns, such as excessive trunk displacement during reaching tasks, should be automatically detected and quantified [32], as these behaviors can impede long-term recovery if left unaddressed. Additional useful metrics include movement frequency (number of repetitions completed), peak velocity, and task success rates (percentage of targets reached or objectives completed) [35,36].

This information should be presented in a manner that is easily visualizable and actionable by clinicians, such as charts, graphs, and heatmaps, that allow clinicians to quickly grasp key trends and areas of concern. This approach enables clinicians to assess patient progress and make informed clinical decisions without the need to analyze raw data or large data sets. Furthermore, providing comprehensive data analytics allows patients to use the game independently, with clinicians reviewing the session data later, making the tool more versatile and practical for unsupervised rehabilitation sessions.

### 4.7. Precision Medicine and Game Adjustability

The concept of precision medicine can be integrated into rehabilitation through adjustable game parameters, allowing clinicians to tailor interventions to the specific needs of each patient. Personalized game settings ensure that the experience is appropriately challenging, fostering both motivation and optimal motor learning [18], where interventions are customized based on the patient’s unique abilities, conditions, and goals.

Key adjustments include settings like patient positioning (e.g., seated, standing, or lying down), limb involvement, target distances, play area size, and event timing. These customizable features are particularly critical for neurological patients or those in early rehabilitation stages, where nuanced and targeted interventions can significantly enhance recovery outcomes. For example, Lange et al. [31] underscored the value of software that allows clinicians to design tasks with ease, an approach that, while ambitious for VR and AR, highlights the importance of enabling user-friendly personalization in these advanced tools.

Game design should strike a balance between simplicity and depth. Quick, generic difficulty settings should coexist with more granular customization options for clinicians with the expertise and time to fine-tune parameters. This ensures both accessibility and precision, meeting diverse clinical needs without overcomplicating the setup process.

Adjustability also supports dynamic difficulty adjustment while maintaining the clinician’s role in refining the challenge level of the exergame. By incorporating Fitts’ law to set movement difficulty, the exergame can adapt to the patient’s progress while remaining optimally challenging and engaging [5]. This dual approach—combining automated adjustments with clinician input—ensures that the exergame remains relevant to the patient’s condition over time and supports personalized, precision-focused care.

### 4.8. Companion Application

A key challenge with delivery systems such as head mounted displays is the potential isolation they create, even in an augmented reality setting where the user can still see their environment. This isolation can be a significant barrier in clinical settings where the clinician needs to monitor the patient’s experience closely [19]. For typical exergames that use body-tracking, the clinician can view the same screen as the patient, allowing them to identify errors or assess the session’s success in real-time.

To address this issue in VR and AR games, a companion application may be helpful. In this instance, it could run on the clinician’s laptop and offer the ability to monitor the patient’s progress, adjust game parameters, and select games without requiring the removal of the headset. This functionality could be delivered via a tablet app or a web-based interface accessible from various devices. The integration of a companion app would make the adoption of VR and AR tools in clinical settings smoother, reducing barriers and enhancing the clinician’s ability to provide real-time feedback and adjustments.

### 4.9. Data Confidentiality and Security

Given the sensitivity of health-related information, it is important to establish a robust software platform that complies with patient data confidentiality and privacy regulations, for example, by keeping patients’ anonymity by using codes instead of identifying information. This includes implementing end-to-end encryption to protect data during transmission and secure storage to prevent unauthorized access. Additionally, role-based access controls should be implemented to ensure that only authorized clinicians can view or modify patient information through these platforms. By prioritizing data security and confidentiality, developers can build trust with both clinicians and patients, which would help better adopt and accept these technologies in healthcare settings.

## 5. Future Directions

### 5.1. Integrating Artificial Intelligence

The integration of artificial intelligence (AI) into rehabilitation exergames offers a promising avenue for enhancing patient outcomes by optimizing data collection and interpretation. AI could also personalize rehabilitation by dynamically adjusting difficulty levels, ensuring that exergames remain both challenging and engaging for patients. AI-driven adaptive algorithms could tailor these experiences based on real-time data and patients’ meaningful areas of interest, thereby maintaining the patient’s motivation and optimizing therapeutic benefits.

Several existing systems demonstrate the potential of AI in rehabilitation contexts. For example, RehaBot, an adaptive serious game platform, uses AI algorithms to assess patients’ range of motion in real-time and automatically adjust game difficulty to match individual capabilities [19]. Similarly, virtual assistants like the MIRA platform guide patients through correct exercise execution and selection [20]. In stroke rehabilitation specifically, AI-powered systems have been integrated with robotics and exoskeletons to provide precise, adaptive assistance, while machine learning models can predict functional recovery trajectories and dynamically adjust therapy intensities based on patient progress [20].

Moreover, AI can revolutionize game navigation, especially for patients with neurological impairments or other impairments limiting their interactions with the games. Traditional input methods like controllers or hand gestures may be too challenging for some of these patients, potentially creating a barrier to their therapy. AI can mitigate this by incorporating alternative navigation methods, such as eye-tracking, as is already done in the Vision pro (Apple, Cupertino, CA, USA), or voice commands, allowing patients to navigate games independently. Even if the game is meant to be played under the supervision of a clinician, this autonomy can significantly enhance a patient’s sense of agency in their rehabilitation process, which has been shown to improve therapeutic outcomes [21].

In addition to improving navigation and engagement, AI’s role in exercise planning and progression cannot be overlooked. By analyzing performance data, AI could recommend specific exercises that are likely to yield the best results for individual patients. For example, if a patient continuously fails to hit a specific target or to move their arm in a specific zone during an exercise, the algorithm could suggest exercises that have been effective for others with similar challenges [31]. This not only enhances the efficacy of the rehabilitation process but also supports clinicians in making data-informed decisions tailored to each patient’s needs. While these AI applications show considerable promise, their integration into clinical practice requires careful validation, addressing concerns about data privacy, algorithm transparency, and the need for continued clinician oversight to ensure therapeutic appropriateness.

### 5.2. Haptic Feedback

Natural haptic feedback, the tactile information received when physically interacting with real objects, plays a critical role in supporting motor learning [5]. However, whether artificial haptic stimulation delivered through devices such as gloves, vibratory controllers, or robotic systems can meaningfully enhance motor learning in post-stroke rehabilitation remains highly debatable [24]. Most current VR systems do not yet accurately incorporate realistic haptic information, and artificial haptics may actually obscure or replace the real haptic feedback that is essential for accurate movement control and sensorimotor learning [37]. This poses a significant challenge, as delivering effective haptic feedback is a critical factor for enhancing both the realism and engagement of augmented reality (AR) games.

While the controllers of current popular AR headsets like the Meta Quest 3 (Meta, Menlo Park, CA, USA) and Apple Vision Pro (Apple, Cupertino, CA, USA) offer a certain degree of haptic feedback through calibrated vibrations, many rehabilitation patients cannot use hand-held controllers, and interactions via controllers lack the natural realism required for fine motor control. Haptic feedback solutions such as haptic gloves or haptic bands are available on the market, but gloves can be encumbering and may actually remove real haptic information by covering the skin and limiting direct tactile contact with objects.

However, the future holds promise for more integrated solutions. One potential avenue lies in the ability of AR to blend real-world objects with augmented reality virtual environments, which could offer more natural forms of haptic feedback [24]. For instance, instead of relying on simulated haptics, patients could interact with real objects within AR-enhanced environments, thus combining the tactile feedback of the real world with the immersive elements of AR. This approach could bridge the gap between physical and digital, enhancing both engagement and therapeutic outcomes. Implementing such a system poses technological challenges, as current mainstream headsets do not yet allow for the direct integration of real-world objects into AR experiences at the level required for rehabilitation games. This would necessitate access to the headset’s camera feed and advanced machine learning models capable of tracking and recognizing objects in real-time. As of now, this possibility is not directly offered to developers, but in the future affordable AR systems could gamify real-world environments for therapeutic purposes.

## 6. Insights from the Living Lab

By facilitating direct interaction between the IRGLM’s hospitalized patients undergoing active rehabilitation and emerging rehabilitation technologies, the Ensemble! Program provides practical insights into how serious games using XR can be integrated into clinical practice to optimize rehabilitation. Through observations and feedback from both clinicians and patients in the technology park, we have identified recurrent challenges in current XR serious games. For example, we noted that patients in the living lab could become frustrated when games were too difficult, which shows the importance of dynamic difficulty adjustment (DDA) and the ability for clinicians to manually customize difficulty. Patients could also become disinterested when games lacked intuitiveness, user-friendliness and positive feedback for correct movements. Interestingly, we observed that patients playing serious games with loss aversion mechanics (gentle constructive penalties for incorrect movements or mistakes) could show greater motivation to correct their movements. Clinicians in the Ensemble! Program have emphasized the need for customizable games that can be tailored to each patient’s rehabilitation needs, goals and capabilities. They also valued tools that can track patient performance, including exercise frequency, quality, consistency, and compensatory movements, ideally with intuitive visualizations, such as charts or graphs. Drawn from our living lab experience, along with motor control, motor learning, and game design principles, these recommendations are intended to contribute to reflections and future developments in the field of rehabilitation XR technologies.

While not a substitute for direct collaboration between developers, clinicians, and patients, these guidelines seek to streamline early interactions and accelerate the development process by providing a common framework for understanding rehabilitation needs. By addressing barriers such as insufficient customization options, inadequate data analytics, and lack of alignment with motor learning principles, developers can create serious games that are more readily adoptable by clinicians and more effective for patient rehabilitation. These recommendations aim to bridge the gap between the rapid advancement of XR technology and the specific requirements of clinical rehabilitation settings, attempting to overcome some of the barriers that have historically hindered the adoption of these technologies in healthcare.

## 7. Limitations

While this commentary article provides general recommendations for the development and adoption of XR technologies in neuromusculoskeletal rehabilitation, several limitations must be acknowledged.

The insights and observations presented are primarily drawn from our team’s experience in a living lab technological park in one setting of the Ensemble! Program, where data collection consisted of participant observations, questionnaire responses, and descriptive analyses (mean values). While innovative, the living lab is not representative of the full diversity of all patient groups and clinical settings and does not provide long-term outcome data. The recommendations might require adaptation for specific patient populations, such as pediatric patients, older adults with cognitive impairments, or individuals with conditions beyond those primarily included in the Ensemble! Program (e.g., patients with stroke, spinal injury, or amputation). Furthermore, while our narrative review synthesized recent XR rehabilitation applications and breakthroughs to inform the design recommendations, it did not employ systematic review methodology or aim for exhaustive coverage of all available literature.

Finally, the rapid pace of technological advancements in XR and AI-driven tools is a challenge for maintaining the relevance of the recommendations. For example, emerging devices such as advanced haptic systems or next-generation AR headsets may introduce new opportunities and challenges not covered in this article. To ensure the continued applicability of these guidelines, iterative updates will be necessary as the technologies evolve.

## 8. Conclusions

The adoption of XR technologies holds significant promise for enhancing physical rehabilitation by increasing patient engagement, providing rich sensory feedback, and enabling intensive, task-specific practice. However, realizing this potential requires careful attention to both the technical and clinical aspects of game design. Through our experience in the Ensemble! Program’s living lab technology park, we observed that while active video games can be well-suited for clinical use, many current and emerging VR and AR applications require further development to include essential features such as clinician customization tools, robust data analytics for monitoring patient progress and movement quality, and further integration of established motor learning principles.

Our commentary synthesizes motor control theory with practical game design through three key contributions: First, translating rehabilitation principles into actionable development recommendations grounded in neuroplasticity, motor learning, and motivational game mechanics such as loss aversion and reward systems [5,14,18]. Second, identifying implementation barriers through direct observation of 20 patients across diverse neuromusculoskeletal conditions in the Ensemble! Program living lab, revealing specific needs such as clinician-customizable difficulty parameters, real-time data analytics, and multimodal feedback systems. Third, providing implementation-focused guidance that bridges theoretical principles (motor control, motor learning, and game theory) with practical clinical features to support adoption across diverse rehabilitation settings [11,12].

While previous work has addressed technical capabilities or clinical theory, our commentary article attempts to synthesize both domains while emphasizing early stakeholder involvement and adaptability to individual patient needs. Our recommendations emphasize the importance of integrating motor control, motor learning and gaming principles, including appropriate challenge calibration, effective extrinsic feedback delivery, and prevention of compensatory movement patterns, with practical clinical requirements such as ease of use, adaptability, and comprehensive data tracking. By incorporating these recommendations early in the development process, developers can create XR serious games that are not only engaging and immersive but also clinically effective and readily adoptable in diverse rehabilitation settings. Continued collaboration among developers, clinicians, researchers, and patients will be essential to refine these technologies and maximize their therapeutic impact for individuals recovering from neuromusculoskeletal impairment.

## Figures and Tables

**Table 1:** Summary of Recommendations for Developing XR Technologies for Neuromusculoskeletal Rehabilitation.

Principle	Recommendation	Purpose	Supporting Evidence
Motivation and Engagement	Incorporate **gamification elements** (e.g., scoring systems, contextual narratives, real-time feedback, rewards).	To enhance patient motivation by making repetitive exercises engaging and rewarding.	Strong [1,2,12,28,29,30]
	Use **loss aversion mechanics** (e.g., penalty systems for mistakes, combo multipliers for correct actions).	To maintain focus, encourage correct, consistent movements, and discourage compensatory behaviors	Moderate [16]
Game Challenge and Dynamic Difficulty	Implement **dynamic difficulty adjustment** (DDA) to automatically calibrate and adapt task difficulty based on patient performance	To ensure tasks remain optimally challenging for motor learning and patient engagement.	Strong [12,13,17]
	Allow clinicians to **manually fine-tune** difficulty settings	To personalize therapy for patients with unique needs or conditions.	Moderate [11,31]
Task Design	Incorporate **adjustable target** sizes, distances, and obstacles.	To encourage precise, coordinated movements and prevent compensatory behaviors.	Strong [32,33]
	Design tasks that emphasize a combination of **movement quality** (KP) and **outcomes** (KR)	To promote proper motor execution and performance-based motivation.	Strong [14,15,34]
Feedback Mechanisms	Provide balanced **positive feedback** (affirming accurate movements) and **negative constructive feedback** (correcting compensatory movements or errors).	To motivate patients, reinforce correct motor patterns and discourage incorrect ones.	Strong [5,15,34]
	Use both **knowledge of performance** (KP) and **knowledge of results** (KR).	To ensure patients understand both how they performed and whether they achieved their goals.	Strong [14,34,35]
	**Avoid** overwhelming patients with **excessive feedback**.	To reduce cognitive overload and encourage intrinsic learning mechanisms.	Strong [5,14,34]
Game Environment	Design games for **specific settings**, such as supervised clinics, home use, or hybrid models.	To ensure games are adaptable to different rehabilitation environments and patient-specific needs in order to optimize clinician supervision, user-friendliness and patient engagement.	Moderate [11,18,31]
Data Analytics and Visualization	Incorporate detailed **data tracking** of patient performance (exercise frequency, quality, and consistency) and identification of compensatory behaviors.	To support data-driven decision-making and personalized therapy adjustments.	Moderate [11,35,36]
	Provide clinicians with clear, intuitive **visualizations** (e.g., charts, graphs, heatmaps).	To facilitate quick assessments of patient progress and areas for improvement.	Moderate [11,36]
Game Customization	Enable **customization** of game parameters (e.g., posture, limb usage, target distances).	To adapt games to various patient conditions and stages of rehabilitation.	Strong [5,13,31,33]
Companion Application	Develop a **companion app** for monitoring, parameter adjustments, and real-time feedback.	To enable clinician oversight and interaction during therapy.	Emerging [31]
Patient Data Privacy and Security	Ensure patient data is **protected** through end-to-end encryption and role-based access controls.	To maintain confidentiality, security and comply with regulations.	Strong (General healthcare standards)
Patient Safety	Include virtual boundaries with real-space alerts, fatigue monitoring (session duration and movement repetition limits), emergency pause functions, and clear exit protocols.	To prevent falls, injuries, and overexertion, particularly during unsupervised or home-based sessions.	Strong [8,9,11,12,34]
AI Integration into Practice	Integrate **AI** for real-time feedback, adaptive difficulty, personalized exercise recommendations, and accessibility tools (e.g., eye-tracking or voice commands).	To optimize therapy by tailoring tasks to patient needs and improving patient autonomy.	Emerging [19,20,21,23]
Future Relevance	**Stay updated** with emerging XR features like advanced haptics and AI-driven adaptivity.	To ensure the technology remains relevant and continues to improve patient outcomes.	Emerging [24,37]

Strong = systematic reviews, meta-analyses, or established motor learning principles; Moderate = scoping reviews, clinical observations, or single studies; Emerging = recent technologies or preliminary research.

## Data Availability

Data collected is not available due to privacy restrictions.

## Abbreviations

The following abbreviations are used in this manuscript:

AI	Artificial Intelligence
AR	Augmented Reality
AVG	Active Video Game
CRIR	Center for Interdisciplinary Research in Rehabilitation of Greater Montreal
DDA	Dynamic Difficulty Adjustment
HMD	Head-Mounted Display
IRGLM	Institut de Réadaptation Gingras-Lindsay de Montréal
IURDPM	Institut Universitaire sur la Réadaptation en Déficience Physique de Montréal
KP	Knowledge of Performance
KR	Knowledge of Results
VR	Virtual Reality
XR	Extended Reality
